# Identifying combinations of long-term conditions associated with sarcopenia: a cross-sectional decision tree analysis in the UK Biobank study

**DOI:** 10.1136/bmjopen-2024-085204

**Published:** 2024-09-05

**Authors:** Susan J Hillman, Richard M Dodds, Antoneta Granic, Miles D Witham, Avan A Sayer, Rachel Cooper

**Affiliations:** 1 AGE Research Group, Translational and Clinical Research Institute, Faculty of Medical Sciences, Newcastle University, Newcastle upon Tyne, UK; 2 NIHR Newcastle Biomedical Research Centre, Newcastle upon Tyne Hospitals NHS Foundation Trust, Cumbria, Northumberland, Tyne and Wear NHS Foundation Trust and Faculty of Medical Sciences, Newcastle University, Newcastle upon Tyne, UK

**Keywords:** aging, epidemiology, chronic disease, machine learning

## Abstract

**Abstract:**

**Objectives:**

This study aims to determine whether machine learning can identify specific combinations of long-term conditions (LTC) associated with increased sarcopenia risk and hence address an important evidence gap—people with multiple LTC (MLTC) have increased risk of sarcopenia but it has not yet been established whether this is driven by specific combinations of LTC.

**Design:**

Decision trees were used to identify combinations of LTC associated with increased sarcopenia risk. Participants were classified as being at risk of sarcopenia based on maximum grip strength of <32 kg for men and <19 kg for women. The combinations identified were triangulated with logistic regression.

**Setting:**

UK Biobank.

**Participants:**

UK Biobank participants with MLTC (two or more LTC) at baseline.

**Results:**

Of 140 001 participants with MLTC (55.3% women, median age 61 years), 21.0% were at risk of sarcopenia. Decision trees identified several LTC combinations associated with an increased risk of sarcopenia. These included drug/alcohol misuse and osteoarthritis, and connective tissue disease and osteoporosis in men, which showed the relative excess risk of interaction of 3.91 (95% CI 1.71 to 7.51) and 2.27 (95% CI 0.02 to 5.91), respectively, in age-adjusted models.

**Conclusion:**

Knowledge of LTC combinations associated with increased sarcopenia risk could aid the identification of individuals for targeted interventions, recruitment of participants to sarcopenia studies and contribute to the understanding of the aetiology of sarcopenia.

**STRENGTHS AND LIMITATIONS OF THIS STUDY**

- We have used decision tree analyses to enable us to identify associations between different combinations of long-term conditions (LTC) and the presence of sarcopenia, without the need for a priori hypotheses.
- By using data from UK Biobank, we have included a large sample of people with multiple LTC (MLTC) across a range of ages in mid-life and later life and have been able to examine many of the LTC that it is now recommended be used to define MLTC.
- Our approach combines the interpretability of individual decision tree analyses with the improved robustness of machine learning ensemble methods and is robust under triangulation with logistic regression.
- The list of LTC included in our analyses is provided for transparency and was selected using guidance from an international Delphi consensus study. However, we acknowledge that there may be other LTC, not included in our analyses that have clinically important associations with sarcopenia.

## Introduction

 The concept of machine learning, as a collection of algorithmic approaches for finding patterns in data, with a view to modelling systems and predicting behaviour, was established in the 1950s.^1^ Machine learning has since been applied in many different industries and areas of academic research including epidemiology.^1^

Of the many different machine learning approaches, decision trees have been specifically identified as having potential value in epidemiological studies because they are ‘a useful tool for identifying homogeneous subgroups defined by combinations of individual characteristics’.^2^ Furthermore, decision trees offer the advantage of interpretability over some other machine learning techniques in that their output can be converted into a set of if-then rules which can be readily understood and sense-checked.^3^ This provides further justification for their use in epidemiology. Despite this, however, there are very few examples of the application of decision tree analyses in epidemiology. To address this gap and assess whether decision trees can be applied to address real-world epidemiological challenges, we aimed to use decision trees to identify groups of long-term conditions (LTC) associated with an increased risk of sarcopenia in a sample of individuals living with multiple LTC (MLTC).

Sarcopenia is defined as a generalised and progressive disorder of skeletal muscle involving accelerated loss of muscle mass and function.^4 5^ Its relatively high prevalence in older populations and relationships with myriad adverse clinical outcomes,^4 5^ including long-term disability,^6^ walking ability,^7^ premature mortality,^8^ mental health disorders^9^ and poorer quality of life^10^ mean that the impacts of sarcopenia are wide-ranging and costly, both in human terms and for health services.^11^ Effective diagnostic strategies are, therefore, required to ensure prevention and treatment can be targeted towards individuals at higher risk of sarcopenia and its deleterious consequences. Methods to define, detect and diagnose sarcopenia have been developed through consensus approaches,^12 13^ including the widely cited European Working Group on Sarcopenia in Older People 2 (EWGSOP2) revised guidelines in 2019^14^ which recommends defining probable sarcopenia based on low muscle strength indicated either by low grip strength or slow chair rise speed.

Multiple long-term conditions (MLTC), also known as multimorbidity, is defined as the presence of two or more LTC^15^ and has been steadily increasing in prevalence over recent decades.^16^ There is a growing body of evidence that individuals living with MLTC, and specifically a greater number of LTC, are at increased risk of sarcopenia,^17^,^21^ but typically these studies treat all LTC equally despite evidence that certain conditions pose a greater risk of sarcopenia, such as diabetes^22^ and osteoarthritis (OA).^23^ By definition, MLTC presents as a combination of conditions within the affected individual, and some combinations are observed more frequently within populations.^16 24^

Given our current knowledge of the relationships between MLTC, specific conditions and sarcopenia, an important next step is to identify specific combinations of LTC which at an individual level are associated with an increased risk of sarcopenia. Such an approach could be beneficial because sarcopenia is not yet routinely assessed in clinical practice and this presents difficulty in the targeting of existing treatments (such as resistance exercise training), and the identification of individuals who are eligible for sarcopenia trials. Information on high-risk combinations of LTC could, therefore, be used to identify individuals who may require assessment for sarcopenia, and hence address these challenges.

Using data from UK Biobank, this study aimed to explore the potential for decision tree analysis to address the real-world challenge of identifying specific combinations of LTC that are associated with an increased risk of sarcopenia in people living with MLTC.

## Methods

### Sample

The UK Biobank study comprises health, lifestyle, genetic and socioeconomic data on ~500 000 participants aged 37–73 years at the time of recruitment across England, Scotland and Wales between 2006 and 2010.^25^

### Assessment of grip strength

Hand grip strength was measured during the baseline assessment according to a standardised protocol using a Jamar hydraulic dynamometer. A single trial was conducted for each hand with the participant seated with forearms supported. We classified participants as ‘at risk’ of sarcopenia by applying cut-points to the maximum grip strength achieved. We used cut points of 2 SD below mean reference values for young adults (<32 kg for men and <19 kg for women)^26^ because these correspond well with values shown to discriminate mobility limitations associated with sarcopenia and have been used in previous work.^27^ This classification therefore comprises people with sarcopenia as well as those considered to have an increased probability of developing sarcopenia at a later time. Participants unable to perform the grip strength tests for health reasons were also placed in this category (n=162).

### Assessment of LTC

During their baseline assessment, participants indicated via touchscreen questionnaire if they had ever been told by a doctor that they had any serious illness or disability, including one or more of the following specific medical conditions: heart attack, angina, stroke, high blood pressure, blood clot in the leg, blood clot in the lung, emphysema/chronic bronchitis, asthma, diabetes or cancer. Those who responded positively were then interviewed by a research nurse who recorded details of all LTC against a hierarchical tree of more than 450 conditions. We assigned each participant a binary variable corresponding to the presence or absence of one or more diagnoses in 53 categories of LTC (online supplemental table 1). Our categories were derived from the classification proposed by Ho *et al*^15^ which describes 24 conditions which should always be included when measuring MLTC, and 35 conditions which should usually be included. Data were not available for six of the conditions which Ho *et al* suggest should usually be included (see online supplemental table 1 for details). We also excluded myositis/myopathy, dermatopolymyositis, dermatomyositis and polymyositis from the connective tissue disease category in line with the usual practice of excluding primary disorders of muscle in sarcopenia studies and trials^28 29^ because they present with low muscle strength not attributable to sarcopenia.

### Statistical analyses

We created decision trees to predict the classification of ‘at risk’ or ‘not at risk’ of sarcopenia from the diagnosed conditions using cross-sectional data collected during the UK Biobank baseline assessment. The trees were configured by iteratively selecting the condition giving the optimum split for detection of the ‘at risk’ classification. Because of the marked imbalance between ‘not at risk’ and ‘at risk’ cases, we used a loss matrix to penalise false negative classifications (‘at risk’ cases misclassified as ‘not at risk’) more heavily than false positives. This was calculated directly from the proportions of the two outcomes. Our model hyperparameters were a maximum depth of 10, a minimum node size of 25 and Gini measure of impurity. We adopted an ensemble approach by creating 1000 decision trees using bootstrap data sets to mitigate the potential for high variance, that is, high dependency on training data. We then analysed the trees and collated the resultant combinations of conditions resulting in an ‘at-risk’ classification, but disregarding those elements of the combination requiring a negative diagnosis for any LTC. We assessed these combinations by summing the probability of a correct classification across the ensemble to yield a composite score which encompassed both the frequency with which the combination appeared and its classification accuracy.

We restricted the sample to participants with MLTC (≥2 LTC), and whose risk of sarcopenia could be classified at baseline. We also stratified all analyses by sex because of recognised differences in patterns of MLTC observed between men and women.^30 31^ Differences between men and women were analysed by using the Mann-Whitney U test for age as a continuous variable, and χ^2^ tests were used for all other variables.

The decision tree analysis resulted in a list of combinations which we ordered by composite score. We retained the 12 combinations with the highest composite scores for each sex as a representative sample of the optimum combinations discovered by the decision trees and then triangulated using logistic regression to assess the degree to which these alternative approaches harmonise; estimate the magnitude of the association of each combination with risk of sarcopenia and explore potential interactions. We calculated the odds ratio (OR) of sarcopenia risk for each condition within each combination as well as interaction terms where relevant. We also estimated the relative excess risk of interaction (RERI) on the additive scale for the combinations comprising two specified conditions. This has been shown to be the most appropriate approach for the assessment of the additional burden of disease solely due to interaction. Within strata associations of each condition in the two combination conditions were also calculated to provide an estimate of the effect modification of each condition on the other.^32^

### Sensitivity analyses

We interrogated the robustness of findings by repeating the decision tree analysis with the three following changes. First, we used the EWGSOP2^14^ grip strength thresholds to determine probable sarcopenia (<27 kg for men and <16 kg for women). Second, we used loss matrices in which the penalty for false negative classifications was doubled. Third, we removed hypertension, the most prevalent condition, to explore if it was a dominant effect.

We also reran our logistic regression models with the inclusion of age as a continuous variable to investigate its role as a potential confounder.

All analyses were conducted by using R V.4.1.2^33^ and we created decision trees using the rpart package, V.4.1–15.^34^

### Patient and public involvement

Patients and/or the public were not involved in this retrospective study.

## Results

A total of 140 001 UK Biobank participants (55.3% women) whose sarcopenia risk could be classified at baseline and who had two or more LTC were included in the analysis. Median age was 62 years (range: 38–72) for men and 61 (range: 40–71) for women, and mean grip strength was 39.3 kg (SD: 9.3) for men and 23.2 (SD: 6.6) for women with 18.2% men and 23.2% women being assessed as at risk of sarcopenia. Prevalence of LTC ranged from 0.4% for metastatic cancers to 59.2% for hypertension. The maximum number of LTC for any individual participant was 14. Tables1  2 show the characteristics of the sample, which showed statistically significant differences by sex (p<0.05) for age, risk of sarcopenia and all but the following four LTC: metastatic cancers, bipolar disorder, post-traumatic stress disorder and inflammatory bowel disease.

**Table 1:** Baseline characteristics of the UK Biobank participants included in the analytical sample by sex*

Characteristic	Females (n=77 488)	Males (n=62 513)	P value†
Age (years)	61 (55–65)	62 (56–66)	<0.001
Age bands (years)			<0.001
38–45	4757 (6.14)	3308 (5.29)	
46–50	6685 (8.63)	4701 (7.52)	
51–55	10 260 (13.24)	7394 (11.83)	
56–60	16 141 (20.83)	11 958 (19.13)	
61–65	23 080 (29.79)	19 510 (31.21)	
66–72	16 565 (21.38)	15 642 (25.02)	
Grip strength (kg)	23.24 (6.62)	39.31 (9.26)	<0.001
At risk of sarcopenia‡	18012 (23.24)	11 386 (18.21)	<0.001
Number of LTC			0.001
2	47 060 (60.73)	38 414 (61.45)	
3	19 395 (25.03)	15 468 (24.74)	
4	7209 (9.3)	5752 (9.2)	
5	2465 (3.18)	1935 (3.1)	
6+	1359 (1.75)	944 (1.51)	

Values shown are n (%), mean (standard deviationSD) or median [(interquartile rangeIQR]).

* Participants were included in the study if they had two two or more long-term conditions and a valid grip strength measure or were unable to perform the test for health reasons, at baseline.Differences between men and women analysed using the Mann-Whitney U test for age as a continuous variable, and chi-squared tests for all other variables.

† Differences between men and women were analysed using the Mann-Whitney U test for age as a continuous variable and χ2 tests for all other variables.Grip strength cut-off values for at risk of sarcopenia classification: for women and for men.

‡ Participants were included in the study if they had 2 two or more long term conditions and a valid grip strength measure, or were unable to perform the test for health reasons, at baseline.Grip strength cut-off values for ‘at risk of sarcopenia’ classification: <19 kg for women and <32 kg for men.

LTClong-term condition

**Table 2:** Prevalence of long-term conditions at baseline in the analytical sample by sex

Long-term condition	Females (n=77 488)	Males (n=62 513)	P value*
Stroke	2583 (3.33)	3686 (5.90)	<0.001
Coronary artery disease	5755 (7.43)	12 907 (20.65)	<0.001
Heart failure	109 (0.14)	191 (0.31)	<0.001
Peripheral artery disease	513 (0.66)	507 (0.81)	0.001
Heart valve disorders	1685 (2.17)	1134 (1.81)	<0.001
Arrhythmia	2328 (3.00)	3218 (5.15)	<0.001
Venous thromboembolic disease	5674 (7.32)	3857 (6.17)	<0.001
Aneurysm	15 (0.02)	82 (0.13)	<0.001
Hypertension	40 841 (52.71)	42 008 (67.20)	<0.001
Diabetes	8405 (10.85)	13 396 (21.43)	<0.001
Addison’s disease	100 (0.13)	47 (0.08)	0.003
Thyroid disorders	16 824 (21.71)	3249 (5.20)	<0.001
Chronic obstructive pulmonary disease	4914 (6.34)	4608 (7.37)	<0.001
Asthma	21 287 (27.47)	14 086 (22.53)	<0.001
Bronchiectasis	622 (0.80)	292 (0.47)	<0.001
Parkinson’s disease	218 (0.28)	335 (0.54)	<0.001
Epilepsy	1353 (1.75)	1291 (2.07)	<0.001
Multiple sclerosis	757 (0.98)	260 (0.42)	<0.001
Paralysis	817 (1.05)	970 (1.55)	<0.001
Transient ischaemic attack	707 (0.91)	764 (1.22)	<0.001
Peripheral neuropathy	246 (0.32)	299 (0.48)	<0.001
Chronic primary pain	539 (0.70)	69 (0.11)	<0.001
Solid organ cancers	14962 (19.31)	7866 (12.58)	<0.001
Haematological cancers	731 (0.94)	919 (1.47)	<0.001
Metastatic cancers	36 (0.05)	23 (0.04)	0.456
Melanoma	1406 (1.81)	962 (1.54)	<0.001
Benign cerebral tumours	69 (0.09)	23 (0.04)	<0.001
Dementia	37 (0.05)	54 (0.09)	0.007
Schizophrenia	141 (0.18)	267 (0.43)	<0.001
Depression	12 846 (16.58)	6734 (10.77)	<0.001
Anxiety	3173 (4.09)	1744 (2.79)	<0.001
Bipolar disorder	553 (0.71)	418 (0.67)	0.329
Drug/alcohol misuse	182 (0.23)	516 (0.83)	<0.001
Eating disorder	249 (0.32)	16 (0.03)	<0.001
Post-traumatic stress disorder	138 (0.18)	132 (0.21)	0.180
Connective tissue disease	4886 (6.31)	2016 (3.22)	<0.001
Osteoarthritis	20 676 (26.68)	10 863 (17.38)	<0.001
Long-term musculoskeletal problems due to injury	4361 (5.63)	4566 (7.30)	<0.001
Osteoporosis	5140 (6.63)	727 (1.16)	<0.001
Gout	484 (0.62)	4942 (7.91)	<0.001
Chronic liver disease	1011 (1.30)	970 (1.55)	<0.001
Inflammatory bowel disease	1511 (1.95)	1206 (1.93)	0.794
Chronic pancreatic disease	404 (0.52)	420 (0.67)	<0.001
Peptic ulcer	1761 (2.27)	2372 (3.79)	<0.001
Chronic kidney disease	546 (0.70)	558 (0.89)	<0.001
End-stage kidney disease	99 (0.13)	159 (0.25)	<0.001
Endometriosis	2579 (3.33)		
Anaemia	2984 (3.85)	931 (1.49)	<0.001
Uncorrectable vision problems	3346 (4.32)	3873 (6.2)	<0.001
Meniere’s disease	610 (0.79)	333 (0.53)	<0.001
HIV/AIDS	43 (0.06)	267 (0.43)	<0.001
Tuberculosis	1007 (1.30)	812 (1.30)	0.999
Congenital disease and chromosomal abnormalities	116 (0.15)	113 (0.18)	0.173

n (%).

* Differences between men and women analysed using chi-squared tests.

The decision tree algorithm identified 783 LTC combinations leading to ‘at-risk’ classification for women across the ensemble and 654 for men. Of these, 19 were identified in at least half of the ensemble for women and 21 for men. The 12 top-scoring combinations for each sex, ranked by composite score, are presented in table 3. Connective tissue disease with any other LTC in women was the most commonly occurring combination with a prevalence of 6.3%. Positive predictive values ranged from 24.54% for paralysis with any other condition in men to 58.16% for coronary artery disease with diabetes and uncorrectable vision problems in women, and specificity ranged from 95.43% for connective tissue disease with any other condition for women to 99.96% for women with multiple sclerosis (MS) and osteoporosis (table 3). One combination comprised three conditions: diabetes, uncorrectable vision problems and coronary artery disease in women, and four combinations comprised only one identified condition, and thus represent the presence of that condition in combination with any of the others. These were connective tissue disease in both men and women, paralysis in men and osteoporosis in men.

**Table 3:** Prevalence, positive predictive value, sensitivity, specificity and accuracy of the 12 LTC combinations most strongly associated with risk of sarcopenia for men and women according to composite score

Rank by composite score*		Prevalence (%)	Positive predictive value	Sensitivity(%)	Specificity (%)	Accuracy(%)
	Women					
1	Multiple sclerosis and osteoporosis	0.06	50.00	0.14	99.96	76.76
2	Connective tissue disease and any other LTC	6.31	44.39	12.04	95.43	76.05
3	COPD and stroke	0.23	50.28	0.50	99.85	76.76
4	Coronary artery disease and osteoarthritis	1.85	41.96	3.33	98.60	76.46
5	Hypertension and multiple sclerosis	0.32	41.22	0.56	99.76	76.70
6	Osteoarthritis and osteoporosis	1.56	41.84	2.80	98.82	76.50
7	Diabetes and osteoarthritis	2.14	40.85	3.75	98.35	76.36
8	Coronary artery disease and diabetes and uncorrectable vision problems	0.13	58.16	0.32	99.93	76.78
9	Connective tissue disease and osteoporosis	0.54	56.63	1.30	99.70	76.83
10	Diabetes and gout	0.15	43.36	0.27	99.89	76.74
11	Chronic primary pain and osteoarthritis	0.22	53.45	0.52	99.86	76.77
12	Epilepsy and venous thromboembolic disease	0.12	43.33	0.22	99.91	76.74
	Men					
1	Connective tissue disease and any other LTC	3.22	35.37	6.26	97.45	80.84
2	Osteoarthritis and stroke	0.61	38.90	1.31	99.54	81.65
3	Connective tissue disease and diabetes	0.41	43.02	0.97	99.71	81.73
4	Diabetes and stroke	1.15	37.02	2.34	99.11	81.49
5	Diabetes and uncorrectable vision problems	1.83	33.77	3.39	98.52	81.19
6	Drug and alcohol misuse and osteoarthritis	0.10	50.00	0.27	99.94	81.79
7	Paralysis and any other LTCs	1.55	24.54	2.09	98.57	81.00
8	Osteoporosis and any other LTCs	1.16	30.81	1.97	99.02	81.34
9	Epilepsy and Stroke	0.30	36.02	0.59	99.77	81.70
10	Connective tissue disease and osteoporosis	0.10	54.69	0.31	99.94	81.80
11	Coronary artery disease and stroke	1.52	33.16	2.77	98.76	81.27
12	Diabetes and osteoarthritis	2.41	30.66	4.06	97.96	80.85

* Combinations are presented in rank order of decreasing composite score. Higher ranking combinations indicates greater combined probability and frequency across the decision tree ensemble.

COPDchronic obstructive pulmonary diseaseLTClong-term condition

Logistic regression revealed that all of the conditions identified in the top 12 combinations were individually associated with increased odds of sarcopenia risk with the exception of uncorrectable vision problems and drug/alcohol misuse (online supplemental tables 2 and 3). Uncorrectable vision problems appear in two combinations: with diabetes in men, and with diabetes and coronary artery disease in women. The RERI for the two combination conditions were in the range 0.36 (95% CI 0.09 to 0.65) for diabetes and OA in men to 3.32 (95% CI 1.43 to 6.38) for drug/alcohol misuse and OA in men and were statistically significant in 8 combinations for women and 7 for men (online supplemental table 2). This indicates that over half of the top 12 combinations identified by decision tree analysis were synergistic. For example, for the combination of drug/alcohol misuse and OA in men which had the highest estimated RERI, the OR of sarcopenia risk for drug/alcohol misuse with OA absent was 1.11 (95% CI 0.87 to 1.4), for OA with drug/alcohol misuse absent it was 1.31 (95% CI 1.25 to 1.38) but when both OA and drug/alcohol misuse were present, the OR was much higher: 4.74 (95% CI 2.88 to 7.8) (online supplemental table 2).

Sensitivity analysis using the EWGSOP2^14^ grip strength thresholds for classification of ‘at risk’ identified 7 of the same top 12 combinations for women and 8 for men. When the analyses were repeated with adjusted loss matrices, 5 of the top 12 combinations were the same for women and 8 were the same for men. With hypertension omitted, 11 of the top 12 combinations were the same for women and 10 were the same for men (online supplemental tables 4–6).

When the logistic regression models were rerun with age, almost all of the associations remained and conclusions were largely unchanged (online supplemental tables 7 and 8).

## Discussion

This study has identified specific combinations of LTC which, in the subgroup of the UK Biobank participants with MLTC at baseline, are more strongly associated with an increased risk of sarcopenia than unspecified combinations of at least two LTC. Among the 12 most strongly associated combinations for each sex, we found 8 showing synergistic effects in women and 7 in men as indicated by the RERI. This means that the number of individuals in this sample who are at risk of sarcopenia is greater than we would expect from the addition of the relative risks for the individual conditions.

Among the combinations identified for each sex, the most commonly occurring LTC were diabetes which is included in three combinations for women and four for men, and OA which is included in four combinations for women and three for men. Other LTC featuring with higher frequency are osteoporosis, connective tissue disease, and stroke, each of which is included in 5 combinations across the 24. This is consistent with findings from previous studies using UK Biobank data. For example, Dodds *et al*^17^ reported cross-sectional associations between a musculoskeletal/trauma category which included OA and osteoporosis and an endocrine conditions category including diabetes with increased odds of probable sarcopenia. Hurst *et al*^18^ also reported positive associations between specific LTC, including some identified in this study, with increased odds of having stable low or declining grip strength over 9 years of follow-up.

Most combinations were robust under sensitivity analysis, which demonstrates the utility of the decision trees in this context despite the exclusion of age in the training data. There were two combinations in which the RERI changed signs in the sensitivity models, one of which included hypertension. This most likely reflects the complexity inherent in including hypertension which is very prevalent among people in mid-life and later life and which has been shown to be negatively associated with sarcopenia in some studies^35^ but the opposite in other studies.^36^ The other combination showing a change with adjustment for age was diabetes and uncorrectable vision problems. Uncorrectable vision problems are a well-known consequence of long-standing or poorly controlled diabetes and in this instance, therefore, it is likely a proxy for the severity of diabetic disease and/or time since diabetes onset.

This study adds important new insights because we have identified specific combinations of LTC associated with an increased risk of sarcopenia among a population, all of whom have MLTC and so are already at increased risk compared with those without MLTC. Furthermore, some of these combinations exhibit a significant synergy, as indicated by the RERI, which has been established by Rothman *et al*^37^ to be the most appropriate means of evaluating the effect of interaction in the public health context. Overall, the seven combinations of LTC for women and six for men which yielded statistically significant additive interactions between the two LTC warrant further investigation because potentially synergistic effects may provide important insights into underlying aetiological mechanisms. Such mechanisms are likely to be specific to the combination in question. For example, OA and alcohol use disorder are both associated with increased levels of inflammatory cytokines^38 39^ which are elevated in people with sarcopenia^40^ and are known to suppress muscle synthesis.^41^ Some combinations, however, such as those including connective tissue disease, and MS with osteoporosis, are likely driving sarcopenia, at least in part, via reduced mobility and disuse atrophy, but connective tissue disease comprises a range of LTC (online supplemental table 1) not all of which share a common mechanism. Further work would therefore be beneficial to explore distinctions between the influences of different connective tissue diseases. Nonetheless, the decision tree approach has found combinations of LTC with a potentially clinically significant synergistic effect; and in so doing not only uncovers clinically useful empirical associations, but may also have generated hypotheses for causal mechanisms for further experimental investigation.

The outputs of all the decision tree sensitivity analyses concur with the main analysis with respect to connective tissue disease with any other LTC, coronary artery disease with OA and diabetes with OA in women; and connective tissue disease with any other LTC, OA with stroke, connective tissue disease with diabetes and epilepsy with stroke in men. These combinations are therefore robust but where differences are noted here these may indicate ways in which future analyses may be refined to respond to more focused clinical questions. For example, the new combinations arising from the analysis using the lower EWGSOP2 grip strength cut-offs may be more pertinent to those with severe grip strength deficits. Similarly, the penalty for false negatives is set arbitrarily, but the sensitivity analysis where this is doubled orients the algorithm towards combinations where fewer ‘at-risk’ cases are missed at the expense of more misclassified ‘not at risk’ cases. This effect could be leveraged depending on the perceived risk of misclassification, for example, screening patients for further investigation versus recruiting for clinical trials on sarcopenic participants.

A strength of our study is the utilisation of data from UK Biobank which, despite its healthy volunteer bias,^42^ includes a large sample of people with MLTC across a range of ages in mid-life and later life. Another strength and novel aspect of this study is that to the authors’ knowledge, the adapted ensemble decision tree approach used has not previously been applied in this context.

Another feature of our approach is that it does not require a priori decisions on the nature of potential effects, which can be seen from the presence of combinations both with and without statistically significant interactions in the output, and the identification of conditions not previously associated with sarcopenia. It, therefore, offers the advantage of being data driven with the potential to uncover unanticipated outcomes.

Our approach is also able to identify combinations when there are many candidate LTC, and this strength should not be underestimated. For example, there are 1378 possible combinations of two, and 23 426 possible combinations of three LTC, using the 53 input LTC in this study; and our decision trees explored potential combinations of up to 10 LTC giving a theoretical number of possible combinations of more than 10^10^. An exhaustive examination of each of these using logistic regression would, therefore, be time-consuming and cause major concerns because of multiple tests.

When compared with other machine learning approaches, the output from decision trees has the advantage of interpretability, which means they can be sense-checked by human experts and the resultant rules can be translated out of the purely computational context. A potential disadvantage, however, is sensitivity to training data. Ensemble techniques are a strategy for mitigating this, but in their usual implementation, they destroy interpretability which is a key outcome for our study. We have therefore used an adapted approach to preserve this and enabled us to examine combinations and discount less clinically relevant findings such as the absence of specific conditions.

Finally, our sensitivity analyses did not yield identical results to our main analyses, which reflects the fact that decision tree models are sensitive to algorithmic choices such as loss matrix values, as well as data choices such as the grip strength threshold for ‘at risk’ whereby using a single set of decision tree hyperparameters is a potential limitation. However, a number of combinations of LTC were identified consistently and findings from decision tree analyses were supported by logistic regression models. Other choices are also likely to influence results, for example, the choice and grouping of input conditions. Although we consider it a strength of our work to have used the list of LTC identified by an international Delphi consensus study by Ho *et al*^15^ as this ensures consistency with other MLTC research, we do acknowledge that as this list is not fully exhaustive there may be other LTC associated with sarcopenia that were not included in our analyses. When implementing this method care is needed with algorithmic decision making to minimise sensitivity to data and ensure optimum performance. Critical examination of decision tree outputs is also required, such as the interaction and sensitivity analyses presented in this study, to enhance understanding of results, eliminate any potentially confounded outputs and support meaningful interpretation.

## Conclusion

Our analysis has demonstrated that decision trees can be used to identify specific combinations of LTC which are consistently associated with an increased risk of sarcopenia in UK Biobank participants with MLTC at baseline, as defined using a standard list of conditions identified in an international Delphi consensus study.^15^ Our findings suggest that people living with MLTC who have specific combinations of LTC may be at higher risk of sarcopenia and hence could be suitable targets for muscle function assessments to improve case finding of sarcopenia, and also for interventions to prevent or mitigate the effects of sarcopenia. An understanding of these combinations of LTC could also inform recruitment to sarcopenia research studies to enhance clinical understanding of the risk factors and the underlying mechanisms for the development of sarcopenia.

## supplementary material

10.1136/bmjopen-2024-085204 online supplemental file 1

## Data Availability

Data may be obtained from a third party and are not publicly available.
