# Supplementary material for: Identifying combinations of long-term conditions associated with sarcopenia: a cross-sectional decision tree analysis in the UK Biobank study
Source: BMJ Open. 2024 Sep 5;14(9):e085204. doi: 10.1136/bmjopen-2024-085204 (PMC11381693; doi:10.1136/bmjopen-2024-085204)
Supplement: online supplemental file 1 [file bmjopen-14-9-s001.pdf]

Supplementary Material for:

Identifying combinations of long-term conditions associated with sarcopenia: a cross-sectional decision tree analysis in the UK Biobank study

Supplementary table 1: Mapping of UK Biobank self-reported conditions at baseline to conditions of Ho et al. BMJ Medicine 2023 http://dx.doi.org/10.1136/bmjmed-2022-000247

Condition from Ho et al [13]	UK Biobank illnesses (fields 20002 and 20001)
Stroke	stroke<br>ischaemic stroke<br>subarachnoid haemorrhage<br>brain haemorrhage<br>subdural haemorrhage/haematoma
Coronary artery disease	heart attack/myocardial infarction<br>angina
Heart failure	heart failure/pulmonary oedema
Peripheral artery disease	peripheral vascular disease<br>leg claudication/ intermittent claudication<br>arterial embolism
Heart valve disorders	mitral valve disease<br>mitral valve prolapse<br>mitral stenosis<br>mitral regurgitation / incompetence<br>aortic valve disease<br>aortic stenosis<br>aortic regurgitation / incompetence<br>heart valve problem/heart murmur
Arrhythmia	svt / supraventricular tachycardia<br>atrial fibrillation<br>atrial flutter<br>heart arrhythmia<br>sick sinus syndrome<br>wolff parkinson white / wpw syndrome<br>irregular heart beat
Venous thromboembolic disease	venous thromboembolic disease<br>pulmonary embolism +/- dvt<br>deep venous thrombosis (dvt)
Aneurysm	aortic aneurysm<br>aortic aneurysm rupture<br>aortic dissection
Hypertension	hypertension<br>essential hypertension
Diabetes mellitus	diabetes<br>type 1 diabetes<br>type 2 diabetes
Addisons disease	adrenocortical insufficiency/addison's disease
Cystic fibrosis	Not present in UK Biobank
Thyroid disorders	hypothyroidism/myxoedema<br>hyperthyroidism/thyrotoxicosis<br>grave's disease<br>thyroiditis<br>thyroid problem (not cancer)<br>thyroid goitre<br>thyroid radioablation therapy
Chronic obstructive pulmonary disease	emphysema/chronic bronchitis<br>bronchitis<br>emphysema<br>chronic obstructive airways disease/copd<br>alpha-1 antitrypsin deficiency
Asthma	asthma

Bronchiectasis	bronchiectasis
Parkinsons	parkinsons disease
Epilepsy	epilepsy
Multiple sclerosis	multiple sclerosis
Paralysis	cerebral palsy<br>paraplegia<br>motor neurone disease<br>spinal cord disorder<br>spina bifida<br>spinal injury<br>acute infective polyneuritis/guillain-barre syndrome<br>polio / poliomyelitis
TIA	transient ischaemic attack (tia)
Peripheral neuropathy	peripheral neuropathy
Chronic primary pain	fibromyalgia
Solid organ cancers	lung cancer<br>breast cancer<br>skin cancer<br>cancer of lip/mouth/pharynx/oral cavity<br>salivary gland cancer<br>larynx/throat cancer<br>nasal cavity cancer<br>ear cancer<br>sinus cancer<br>lip cancer<br>tongue cancer<br>gum cancer<br>parotid gland cancer<br>other salivary gland cancer<br>oesophageal cancer<br>stomach cancer<br>small intestine/small bowel cancer<br>large bowel cancer/colorectal cancer<br>anal cancer<br>colon cancer/sigmoid cancer<br>rectal cancer<br>liver/hepatocellular cancer<br>gallbladder/bile duct cancer<br>pancreas cancer<br>small cell lung cancer<br>non-small cell lung cancer<br>peripheral nerve/autonomic nerve cancer<br>eye and/or adnexal cancer<br>meningeal cancer/malignant meningioma<br>brain cancer/primary malignant brain tumour<br>spinal cord or cranial nerve cancer<br>kidney/renal cell cancer<br>bladder cancer<br>other cancer of urinary tract<br>female genital tract cancer<br>male genital tract cancer<br>ovarian cancer<br>uterine/endometrial cancer<br>cervical cancer<br>vaginal cancer<br>vulval cancer<br>prostate cancer<br>testicular cancer

	penis cancer<br>non-melanoma skin cancer<br>basal cell carcinoma<br>squamous cell carcinoma<br>primary bone cancer<br>mesothelioma<br>thyroid cancer<br>parathyroid cancer<br>adrenal cancer<br>sarcoma/fibrosarcoma<br>malignant lymph node, unspecified<br>cin/pre-cancer cells cervix<br>rodent ulcer<br>retinoblastoma<br>kaposis sarcoma<br>mouth cancer<br>tonsil cancer<br>oropharynx/oropharyngeal cancer<br>trachea cancer<br>thymus cancer/malignant thymoma<br>heart/mediastinum cancer<br>respiratory/intrathoracic cancer<br>appendix cancer<br>fallopian tube cancer<br>malignant insulinoma
Haematological cancers	lymphoma<br>hodgkins lymphoma / hodgkins disease<br>non-hodgkins lymphoma<br>leukaemia<br>chronic lymphocytic<br>chronic myeloid<br>acute myeloid leukaemia<br>multiple myeloma<br>other haematological malignancy
Metastatic cancers	metastatic cancer (unknown primary)<br>bone metastases / bony secondaries
Melanoma	malignant melanoma
Benign cerebral tumours	meningioma / benign meningeal tumour
Dementia	dementia/alzheimers/cognitive impairment
Schizophrenia	schizophrenia
Depression	depression
Anxiety	anxiety/panic attacks
Bipolar disorder	mania/bipolar disorder/manic depression
Drug and/or alcohol misuse	alcohol dependency<br>opioid dependency<br>other substance abuse/dependency<br>substance abuse/dependency
Eating disorder	anorexia/bulimia/other eating disorder
Autism	Not present in UK Biobank
PTSD	post-traumatic stress disorder
Connective tissue disease	connective tissue disorder<br>vasculitis<br>giant cell/temporal arteritis<br>polymyalgia rheumatica<br>wegners granulmatosis<br>microscopic polyarteritis<br>polyartertis nodosa<br>systemic lupus erythematosis/sle

	sjogren's syndrome/sicca syndrome<br>scleroderma/systemic sclerosis<br>rheumatoid arthritis<br>raynaud's phenomenon/disease<br>psoriatic arthropathy
Osteoarthritis	osteoarthritis<br>arthritis (nos)
Long-term MSK problems due to injury	spinal injury<br>neck problem/injury<br>prolapsed disc/slipped disc<br>muscle or soft tissue injuries<br>tendonitis / tendinitis / tenosynovitis<br>bursitis<br>housemaid's knee (prepatellar bursitis)<br>epicondylitis<br>tennis elbow / lateral epicondylitis
Osteoporosis	osteoporosis
Gout	gout
Chronic liver disease	alcoholic liver disease / alcoholic cirrhosis<br>liver failure/cirrhosis<br>primary biliary cirrhosis<br>hepatitis<br>infective/viral hepatitis<br>hepatitis a<br>hepatitis b<br>hepatitis c<br>hepatitis d<br>hepatitis e<br>non-infective hepatitis<br>sclerosing cholangitis
Inflammatory bowel disease	crohns disease<br>ulcerative colitis<br>inflammatory bowel disease
Chronic pancreatic disease	pancreatic disease<br>pancreatitis
Peptic ulcer	gastric/stomach ulcers<br>duodenal ulcer
Chronic kidney disease	renal/kidney failure<br>renal failure not requiring dialysis<br>polycystic kidney<br>kidney nephropathy<br>iga nephropathy<br>diabetic nephropathy<br>nephritis<br>glomerulnephritis
End-stage kidney disease	renal failure requiring dialysis
Endometriosis	endometriosis
Chronic urinary tract infection	Not present in UK Biobank
Anaemia	iron deficiency anaemia<br>pernicious anaemia<br>thalassaemia<br>sickle cell disease<br>aplastic anaemia<br>anaemia
Uncorrectable visual impairment	glaucoma<br>retinal problem<br>retinal detachment<br>retinal artery/vein occlusion

	retinitis pigmentosa<br>macular degeneration<br>diabetic eye disease<br>eye trauma<br>optic neuritis
Uncorrectable hearing impairment	Not present in UK Biobank
Menieres disease	meniere's disease
HIV/AIDS	hiv/aids
Chronic Lyme Disease	Not present in UK Biobank
Tuberculosis	tuberculosis (tb)
Long COVID	Not present in UK Biobank
Congenital disease and chromosomal abnormalities	cerebral palsy<br>spina bifida

Supplementary table 2: Odds ratios of sarcopenia risk (including within strata effects) and relative excess risk of interactions [95% CI] for two condition combinations identified in decision tree analyses

2a: Multiple sclerosis and Osteoporosis in women

*	Osteoporosis absent	Osteoporosis present	Effect of Osteoporosis within the strata of Multiple sclerosis
	OR [95% CI]	OR [95% CI]	OR [95% CI]
Multiple sclerosis absent	1 [Reference]	1.57 [1.48, 1.67]	1.57 [1.48, 1.67]
Multiple sclerosis present	1.58 [1.35, 1.85]	3.43 [1.97, 5.98]	2.17 [1.22, 3.86]
Effect of Multiple sclerosis within the strata of Osteoporosis	1.58 [1.35, 1.85]	2.18 [1.25, 3.81]	
Multiplicative scale	1.38 [0.77, 2.47]		
RERI	1.28 [-0.21, 3.83]		

2b: Chronic obstructive pulmonary disease and Stroke in women

*	Stroke absent	Stroke present	Effect of Stroke within the strata of Chronic obstructive pulmonary disease
	OR [95% CI]	OR [95% CI]	OR [95% CI]
Chronic obstructive pulmonary disease absent	1 [Reference]	1.49 [1.37, 1.63]	1.49 [1.37, 1.63]
Chronic obstructive pulmonary disease present	1.17 [1.09, 1.25]	3.43 [2.56, 4.6]	2.93 [2.17, 3.96]
Effect of Chronic obstructive pulmonary disease within the strata of Stroke	1.17 [1.09, 1.25]	2.3 [1.69, 3.12]	
Multiplicative scale	1.96 [1.43, 2.68]		
RERI	**1.77 [0.88, 2.94]**		

2c: Coronary artery disease and Osteoarthritis in women

*	Osteoarthritis absent	Osteoarthritis present	Effect of Osteoarthritis within the strata of Coronary artery disease
	OR [95% CI]	OR [95% CI]	OR [95% CI]
Coronary artery disease absent	1 [Reference]	1.73 [1.67, 1.8]	1.73 [1.67, 1.8]
Coronary artery disease present	1.64 [1.53, 1.76]	2.93 [2.63, 3.26]	1.78 [1.57, 2.02]
Effect of Coronary artery disease within the strata of Osteoarthritis	1.64 [1.53, 1.76]	1.69 [1.51, 1.89]	
Multiplicative scale	1.03 [0.9, 1.17]		
RERI	**0.55 [0.23, 0.90]**		

2d: Hypertension and Multiple sclerosis in women

*	Multiple sclerosis absent	Multiple sclerosis present	Effect of Multiple sclerosis within the strata of Hypertension
	OR [95% CI]	OR [95% CI]	OR [95% CI]
Hypertension absent	1 [Reference]	1.39 [1.14, 1.68]	1.39 [1.14, 1.68]
Hypertension present	1.07 [1.03, 1.11]	2.41 [1.87, 3.12]	2.26 [1.75, 2.91]
Effect of Hypertension within the strata of Multiple sclerosis	1.07 [1.03, 1.11]	1.74 [1.27, 2.39]	
Multiplicative scale	1.63 [1.18, 2.24]		
RERI	**0.96 [0.34, 1.70]**		

2e: Osteoarthritis and Osteoporosis in women

*	Osteoporosis absent	Osteoporosis present	Effect of Osteoporosis within the strata of Osteoarthritis
	OR [95% CI]	OR [95% CI]	OR [95% CI]
Osteoarthritis absent	1 [Reference]	1.6 [1.49, 1.72]	1.6 [1.49, 1.72]
Osteoarthritis present	1.73 [1.67, 1.8]	2.89 [2.58, 3.25]	1.67 [1.48, 1.88]
Effect of Osteoarthritis within the strata of Osteoporosis	1.73 [1.67, 1.8]	1.81 [1.58, 2.07]	
Multiplicative scale	1.04 [0.91, 1.2]		
RERI	**0.56 [0.22, 0.93]**		

2f: Diabetes and Osteoarthritis in women

*	Osteoarthritis absent	Osteoarthritis present	Effect of Osteoarthritis within the strata of Diabetes
	OR [95% CI]	OR [95% CI]	OR [95% CI]
Diabetes absent	1 [Reference]	1.74 [1.67, 1.8]	1.74 [1.67, 1.8]
Diabetes present	1.45 [1.36, 1.53]	2.81 [2.54, 3.11]	1.94 [1.74, 2.17]
Effect of Diabetes within the strata of Osteoarthritis	1.45 [1.36, 1.53]	1.62 [1.46, 1.79]	
Multiplicative scale	1.12 [0.99, 1.26]		
RERI	**0.63 [0.35, 0.93]**		

2g: Connective tissue disease and Osteoporosis in women

*	Osteoporosis absent	Osteoporosis present	Effect of Osteoporosis within the strata of Connective tissue disease
	OR [95% CI]	OR [95% CI]	OR [95% CI]
Connective tissue disease absent	1 [Reference]	1.54 [1.44, 1.64]	1.54 [1.44, 1.64]
Connective tissue disease present	2.82 [2.65, 3]	4.83 [3.97, 5.86]	1.71 [1.4, 2.1]
Effect of Connective tissue disease within the strata of Osteoporosis	2.82 [2.65, 3]	3.14 [2.56, 3.85]	
Multiplicative scale	1.11 [0.9, 1.38]		
RERI	**1.47 [0.59, 2.52]**		

2h: Diabetes and Gout in women

*	Gout absent	Gout present	Effect of Gout within the strata of Diabetes
	OR [95% CI]	OR [95% CI]	OR [95% CI]
Diabetes absent	1 [Reference]	1.53 [1.23, 1.91]	1.53 [1.23, 1.91]
Diabetes present	1.4 [1.33, 1.47]	2.64 [1.82, 3.83]	1.88 [1.29, 2.74]
Effect of Diabetes within the strata of Gout	1.4 [1.33, 1.47]	1.73 [1.12, 2.66]	
Multiplicative scale	1.23 [0.8, 1.9]		
RERI	0.71 [-0.2, 1.94]		

2i: Chronic primary pain and Osteoarthritis in women

*	Osteoarthritis absent	Osteoarthritis present	Effect of Osteoarthritis within the strata of Chronic primary pain
	OR [95% CI]	OR [95% CI]	OR [95% CI]
Chronic primary pain absent	1 [Reference]	1.72 [1.66, 1.78]	1.72 [1.66, 1.78]
Chronic primary pain present	1.79 [1.44, 2.24]	4.47 [3.32, 6.03]	2.5 [1.72, 3.62]
Effect of Chronic primary pain within the strata of Osteoarthritis	1.79 [1.44, 2.24]	2.6 [1.93, 3.51]	
Multiplicative scale	1.45 [1, 2.11]		
RERI	**1.96 [0.73, 3.56]**		

2j: Epilepsy and Venous thromboembolic disease in women

*	Venous thromboembolic disease absent	Venous thromboembolic disease absent present	Effect of Venous thromboembolic disease absent within the strata of Epilepsy
	OR [95% CI]	OR [95% CI]	OR [95% CI]
Epilepsy absent	1 [Reference]	1.15 [1.08, 1.23]	1.15 [1.08, 1.23]
Epilepsy present	1.21 [1.06, 1.37]	2.56 [1.69, 3.89]	2.13 [1.38, 3.29]
Effect of Epilepsy within the strata of Venous thromboembolic disease absent	1.21 [1.06, 1.37]	2.23 [1.46, 3.39]	
Multiplicative scale	1.85 [1.19, 2.87]		
RERI	**1.21 [0.32, 2.54]**		

2k: Osteoarthritis and Stroke in men

*	Stroke absent	Stroke present	Effect of Stroke within the strata of Osteoarthritis
	OR [95% CI]	OR [95% CI]	OR [95% CI]
Osteoarthritis absent	1 [Reference]	1.61 [1.48, 1.75]	1.61 [1.48, 1.75]
Osteoarthritis present	1.32 [1.26, 1.39]	3.12 [2.54, 3.84]	2.36 [1.91, 2.91]
Effect of Osteoarthritis within the strata of Stroke	1.32 [1.26, 1.39]	1.94 [1.56, 2.42]	
Multiplicative scale	1.47 [1.17, 1.84]		
RERI	**1.19 [0.59, 1.91]**		

2l: Connective tissue disease and Diabetes in men

*	Diabetes absent	Diabetes present	Effect of Diabetes within the strata of Connective tissue disease
	OR [95% CI]	OR [95% CI]	OR [95% CI]
Connective tissue disease absent	1 [Reference]	1.64 [1.57, 1.72]	1.64 [1.57, 1.72]
Connective tissue disease present	2.74 [2.48, 3.04]	3.98 [3.11, 5.1]	1.45 [1.11, 1.89]
Effect of Connective tissue disease within the strata of Diabetes	2.74 [2.48, 3.04]	2.42 [1.89, 3.11]	
Multiplicative scale	0.88 [0.67, 1.16]		
RERI	0.59 [-0.32, 1.74]		

2m: Diabetes and Stroke in men

*	Stroke absent	Stroke present	Effect of Stroke within the strata of Diabetes
	OR [95% CI]	OR [95% CI]	OR [95% CI]
Diabetes absent	1 [Reference]	1.6 [1.46, 1.75]	1.6 [1.46, 1.75]
Diabetes present	1.58 [1.51, 1.66]	3.05 [2.62, 3.56]	1.93 [1.65, 2.25]
Effect of Diabetes within the strata of Stroke	1.58 [1.51, 1.66]	1.91 [1.61, 2.27]	
Multiplicative scale	1.21 [1.01, 1.44]		
RERI	**0.87 [0.41, 1.39]**		

2n: Diabetes and Uncorrectable vision problems in men

*	Uncorrectable vision problems absent	Uncorrectable vision problems present	Effect of Uncorrectable vision problems within the strata of Diabetes
	OR [95% CI]	OR [95% CI]	OR [95% CI]
Diabetes absent	1 [Reference]	1.04 [0.93, 1.15]	1.04 [0.93, 1.15]
Diabetes present	1.52 [1.45, 1.6]	2.57 [2.27, 2.91]	1.69 [1.48, 1.92]
Effect of Diabetes within the strata of Uncorrectable vision problems	1.52 [1.45, 1.6]	2.48 [2.12, 2.9]	
Multiplicative scale	1.63 [1.38, 1.92]		
RERI	**1.01 [0.69, 1.36]**		

2o: Drug and/or alcohol misuse and Osteoarthritis in men

*	Osteoarthritis absent	Osteoarthritis present	Effect of Osteoarthritis within the strata of Drug and/or alcohol misuse
	OR [95% CI]	OR [95% CI]	OR [95% CI]
Drug and/or alcohol misuse absent	1 [Reference]	1.31 [1.25, 1.38]	1.31 [1.25, 1.38]
Drug and/or alcohol misuse present	1.11 [0.87, 1.4]	4.74 [2.88, 7.8]	4.28 [2.47, 7.42]
Effect of Drug and/or alcohol misuse within the strata of Osteoarthritis	1.11 [0.87, 1.4]	3.6 [2.19, 5.94]	
Multiplicative scale	3.26 [1.87, 5.66]		
RERI	**3.32 [1.43, 6.38]**		

2p: Epilepsy and Stroke in men

*	Stroke absent	Stroke present	Effect of Stroke within the strata of Epilepsy
	OR [95% CI]	OR [95% CI]	OR [95% CI]
Epilepsy absent	1 [Reference]	1.61 [1.49, 1.74]	1.61 [1.49, 1.74]
Epilepsy present	1.34 [1.16, 1.54]	2.63 [1.95, 3.55]	1.97 [1.41, 2.74]
Effect of Epilepsy within the strata of Stroke	1.34 [1.16, 1.54]	1.63 [1.2, 2.22]	
Multiplicative scale	1.22 [0.87, 1.71]		
RERI	0.68 [-0.04, 1.62]		

2q: Connective tissue disease and Osteoporosis in men

*	Osteoporosis absent	Osteoporosis present	Effect of Osteoporosis within the strata of Connective tissue disease
	OR [95% CI]	OR [95% CI]	OR [95% CI]
Connective tissue disease absent	1 [Reference]	1.88 [1.58, 2.22]	1.88 [1.58, 2.22]
Connective tissue disease present	2.51 [2.28, 2.76]	5.68 [3.47, 9.3]	2.27 [1.37, 3.74]
Effect of Connective tissue disease within the strata of Osteoporosis	2.51 [2.28, 2.76]	3.03 [1.8, 5.09]	
Multiplicative scale	1.21 [0.71, 2.05]		
RERI	**2.3 [0.05, 5.93]**		

2r: Coronary artery disease and Stroke in men

*	Stroke absent	Stroke present	Effect of Stroke within the strata of Coronary artery disease
	OR [95% CI]	OR [95% CI]	OR [95% CI]
Coronary artery disease absent	1 [Reference]	1.53 [1.4, 1.68]	1.53 [1.4, 1.68]
Coronary artery disease present	1.3 [1.23, 1.36]	2.44 [2.13, 2.8]	1.88 [1.63, 2.17]
Effect of Coronary artery disease within the strata of Stroke	1.3 [1.23, 1.36]	1.59 [1.35, 1.87]	
Multiplicative scale	1.23 [1.04, 1.45]		
RERI	**0.61 [0.27, 0.99]**		

2s: Diabetes and Osteoarthritis in men

*	Osteoarthritis absent	Osteoarthritis present	Effect of Osteoarthritis within the strata of Diabetes
	OR [95% CI]	OR [95% CI]	OR [95% CI]
Diabetes absent	1 [Reference]	1.38 [1.31, 1.46]	1.38 [1.31, 1.46]
Diabetes present	1.63 [1.55, 1.72]	2.38 [2.12, 2.66]	1.46 [1.29, 1.64]
Effect of Diabetes within the strata of Osteoarthritis	1.63 [1.55, 1.72]	1.72 [1.52, 1.94]	
Multiplicative scale	1.05 [0.92, 1.2]		
RERI	**0.36 [0.09, 0.65]**		

OR: odds ratio. CI: confidence interval

RERI: Relative excess risk of interaction. The difference between the observed relative risk and the combined relative risk of the two conditions individually on the additive scale.

Supplementary table 3: Odds ratios of sarcopenia risk for those single and three condition combinations identified in decision tree analyses. Estimates shown compare those people with the condition to those people without (ref).

	**OR [95% CI]**
**Women**	
Connective tissue disease	**2.86 [2.70, 3.03]**
Coronary artery disease	**1.57 [1.47, 1.67]**
Diabetes	**1.34 [1.26, 1.42]**
Uncorrectable vision problems	1.05 [0.95, 1.15]
Coronary artery disease and Diabetes	0.99 [0.85, 1.16]
Coronary artery disease and Uncorrectable vision problems	1.18 [0.82, 1.69]
Diabetes and Uncorrectable vision problems	**1.27 [1.04, 1.56]**
Coronary artery disease and Diabetes and Uncorrectable vision problems	1.52 [0.85, 2.74]
**Men**	
Connective tissue disease	**2.55 [2.33, 2.80]**
Paralysis	**1.47 [1.27, 1.70]**
Osteoporosis	**2.02 [1.72, 2.36]**

OR: odds ratio. CI: confidence interval

Supplementary table 4: Top twelve scoring combinations for women and men with 'at risk' classification determined from EWGSOP2 grip thresholds
https://doi.org/10.1093/ageing/afy169

Women	Men
Connective tissue disease and Osteoporosis	Drug/alcohol misuse and Osteoarthritis
Diabetes and Osteoarthritis and Osteoporosis	Connective tissue disease and any other LTC
Connective tissue disease and any other LTC	Epilepsy and Stroke
Asthma and Coronary artery disease and Osteoarthritis	Paralysis and any other LTC
Osteoarthritis and Osteoporosis	Diabetes and Osteoarthritis and Uncorrectable vision problems
Chronic primary pain and Osteoarthritis	Connective tissue disease and Diabetes
Diabetes and Osteoarthritis	Diabetes and Uncorrectable vision problems
Coronary artery disease and Osteoarthritis	Epilepsy and Osteoarthritis
Diabetes and Stroke	Osteoarthritis and Stroke
Chronic primary pain and any other LTC	Depression and Osteoarthritis
Depression and Diabetes and Osteoarthritis	Coronary artery disease and Diabetes and Stroke
Hypertension and Multiple sclerosis	Coronary artery disease and Diabetes and Uncorrectable vision problems

Supplementary table 5: Top twelve scoring combinations for women and men using loss matrices in which the penalty for false negative classification was doubled

Women	Men
Connective tissue disease and any other LTC	Connective tissue disease and any other LTC
Multiple sclerosis and Osteoporosis	Osteoarthritis and Stroke
Osteoarthritis and any other LTC	Connective tissue disease and Diabetes
Coronary artery disease and any other LTC	Diabetes and Uncorrectable vision problems
Osteoporosis and any other LTC	Diabetes and any other LTC
Diabetes and any other LTC	Coronary artery disease and Stroke
Coronary artery disease and Diabetes	Diabetes and Stroke
Stroke and any other LTC	Epilepsy and Stroke
Chronic obstructive pulmonary disease (COPD) and Osteoporosis	Osteoporosis and any other LTC
Coronary artery disease and Osteoarthritis	Stroke and any other LTC
Diabetes and Osteoarthritis	Osteoarthritis and any other LTC
Chronic obstructive pulmonary disease and Stroke	Depression and Osteoarthritis

Supplementary table 6: Top twelve scoring combinations for women and men omitting the most prevalent condition: hypertension

Women	Men
Multiple sclerosis and Osteoporosis	Connective tissue disease and Diabetes
Connective tissue disease and any other LTC	Drug/alcohol misuse and Osteoarthritis
Chronic obstructive pulmonary disease (COPD) and Stroke	Connective tissue disease and any other LTC
Coronary artery disease and Osteoarthritis	Osteoarthritis and Stroke
Osteoarthritis and Osteoporosis	Diabetes and Stroke
Chronic primary pain and Osteoarthritis	Anaemia and Diabetes
Diabetes and Osteoarthritis	Diabetes and Uncorrectable vision problems
Diabetes and Gout	Connective tissue disease and Osteoporosis
Connective tissue disease and Osteoporosis	Connective tissue disease and Coronary artery disease
Coronary artery disease and Diabetes and Uncorrectable vision problems	Osteoporosis and any other LTC
Epilepsy and Venous thromboembolic disease	Epilepsy and Stroke
Chronic obstructive pulmonary disease and Osteoporosis	Paralysis and any other LTC

Supplementary table 7: Odds ratios of sarcopenia risk (including within strata effects) and relative excess risk of interactions [95% CI] for two condition combinations identified in decision tree analyses in age adjusted models

7a: Multiple sclerosis and Osteoporosis in women, model adjusted for age

*	Osteoporosis absent	Osteoporosis present	Effect of Osteoporosis within the strata of Multiple sclerosis
	OR [95% CI]	OR [95% CI]	OR [95% CI]
Multiple sclerosis absent	1 [Reference]	1.36 [1.28, 1.45]	1.36 [1.28, 1.45]
Multiple sclerosis present	1.87 [1.59, 2.2]	3.36 [1.92, 5.88]	1.79 [1, 3.21]
Effect of Multiple sclerosis within the strata of Osteoporosis	1.87 [1.59, 2.2]	2.47 [1.41, 4.34]	
Age	1.05 [1.05, 1.06]		
Multiplicative scale	1.32 [0.73, 2.37]		
RERI	1.12 [-0.36, 3.66]		

7b: Chronic obstructive pulmonary disease and Stroke in women, model adjusted for age

*	Stroke absent	Stroke present	Effect of Stroke within the strata of Chronic obstructive pulmonary disease
	OR [95% CI]	OR [95% CI]	OR [95% CI]
Chronic obstructive pulmonary disease absent	1 [Reference]	1.42 [1.3, 1.55]	1.42 [1.3, 1.55]
Chronic obstructive pulmonary disease present	1.15 [1.08, 1.23]	3.19 [2.37, 4.29]	2.77 [2.04, 3.75]
Effect of Chronic obstructive pulmonary disease within the strata of Stroke	1.15 [1.08, 1.23]	2.25 [1.65, 3.06]	
Age	1.05 [1.05, 1.06]		
Multiplicative scale	1.95 [1.42, 2.68]		
RERI	**1.62 [0.78, 2.73]**		

7c: Coronary artery disease and Osteoarthritis in women, model adjusted for age

*	Osteoarthritis absent	Osteoarthritis present	Effect of Osteoarthritis within the strata of Coronary artery disease
	OR [95% CI]	OR [95% CI]	OR [95% CI]
Coronary artery disease absent	1 [Reference]	1.56 [1.5, 1.62]	1.56 [1.5, 1.62]
Coronary artery disease present	1.4 [1.31, 1.51]	2.45 [2.2, 2.74]	1.75 [1.54, 1.98]
Effect of Coronary artery disease within the strata of Osteoarthritis	1.4 [1.31, 1.51]	1.57 [1.41, 1.76]	
Age	1.05 [1.05, 1.05]		
Multiplicative scale	1.12 [0.98, 1.28]		
RERI	**0.49 [0.22, 0.78]**		

7d: Hypertension and Multiple sclerosis in women, model adjusted for age

*	Multiple sclerosis absent	Multiple sclerosis present	Effect of Multiple sclerosis within the strata of Hypertension
	OR [95% CI]	OR [95% CI]	OR [95% CI]
Hypertension absent	1 [Reference]	2.56 [1.98, 3.32]	2.56 [1.98, 3.32]
Hypertension present	1.04 [1.01, 1.08]	1.7 [1.4, 2.07]	1.63 [1.34, 1.98]
Effect of Hypertension within the strata of Multiple sclerosis	1.04 [1.01, 1.08]	0.66 [0.48, 0.92]	
Age	1.05 [1.05, 1.06]		
Multiplicative scale	0.64 [0.46, 0.88]		
RERI	-0.91 [-1.72, -0.22]		

7e: Osteoarthritis and Osteoporosis in women, model adjusted for age

*	Osteoporosis absent	Osteoporosis present	Effect of Osteoporosis within the strata of Osteoarthritis
	OR [95% CI]	OR [95% CI]	OR [95% CI]
Osteoarthritis absent	1 [Reference]	1.37 [1.27, 1.48]	1.37 [1.27, 1.48]
Osteoarthritis present	1.56 [1.5, 1.62]	2.4 [2.13, 2.7]	1.53 [1.36, 1.73]
Effect of Osteoarthritis within the strata of Osteoporosis	1.56 [1.5, 1.62]	1.75 [1.53, 2]	
Age	1.05 [1.05, 1.05]		
Multiplicative scale	1.12 [0.97, 1.29]		
RERI	**0.46 [0.18, 0.77]**		

7f: Diabetes and Osteoarthritis in women, model adjusted for age

*	Osteoarthritis absent	Osteoarthritis present	Effect of Osteoarthritis within the strata of Diabetes
	OR [95% CI]	OR [95% CI]	OR [95% CI]
Diabetes absent	1 [Reference]	1.57 [1.51, 1.63]	1.57 [1.51, 1.63]
Diabetes present	1.38 [1.3, 1.46]	2.51 [2.27, 2.78]	1.82 [1.62, 2.03]
Effect of Diabetes within the strata of Osteoarthritis	1.38 [1.3, 1.46]	1.6 [1.44, 1.78]	
Age	1.05 [1.05, 1.05]		
Multiplicative scale	1.16 [1.03, 1.31]		
RERI	**0.56 [0.31, 0.84]**		

7g: Connective tissue disease and Osteoporosis in women, model adjusted for age

*	Osteoporosis absent	Osteoporosis present	Effect of Osteoporosis within the strata of Connective tissue disease
	OR [95% CI]	OR [95% CI]	OR [95% CI]
Connective tissue disease absent	1 [Reference]	1.33 [1.24, 1.42]	1.33 [1.24, 1.42]
Connective tissue disease present	2.87 [2.69, 3.05]	4.33 [3.56, 5.28]	1.51 [1.23, 1.86]
Effect of Connective tissue disease within the strata of Osteoporosis	2.87 [2.69, 3.05]	3.26 [2.66, 4.01]	
Age	1.05 [1.05, 1.06]		
Multiplicative scale	1.14 [0.92, 1.41]		
RERI	**1.14 [0.34, 2.1]**		

7h: Diabetes and Gout in women, model adjusted for age

*	Gout absent	Gout present	Effect of Gout within the strata of Diabetes
	OR [95% CI]	OR [95% CI]	OR [95% CI]
Diabetes absent	1 [Reference]	1.35 [1.08, 1.69]	1.35 [1.08, 1.69]
Diabetes present	1.36 [1.29, 1.43]	2.26 [1.55, 3.29]	1.66 [1.14, 2.43]
Effect of Diabetes within the strata of Gout	1.36 [1.29, 1.43]	1.68 [1.08, 2.59]	
Age	1.05 [1.05, 1.06]		
Multiplicative scale	1.24 [0.8, 1.92]		
RERI	0.55 [-0.23, 1.62]		

7i: Chronic primary pain and Osteoarthritis in women, model adjusted for age

*	Osteoarthritis absent	Osteoarthritis present	Effect of Osteoarthritis within the strata of Chronic primary pain
	OR [95% CI]	OR [95% CI]	OR [95% CI]
Chronic primary pain absent	1 [Reference]	1.55 [1.5, 1.61]	1.55 [1.5, 1.61]
Chronic primary pain present	2.05 [1.64, 2.57]	4.92 [3.64, 6.66]	2.39 [1.65, 3.49]
Effect of Chronic primary pain within the strata of Osteoarthritis	2.05 [1.64, 2.57]	3.17 [2.34, 4.29]	
Age	1.05 [1.05, 1.05]		
Multiplicative scale	1.54 [1.06, 2.25]		
RERI	**2.31 [0.93, 4.1]**		

7j: Epilepsy and Venous thromboembolic disease in women, model adjusted for age

*	Venous thromboembolic disease absent	Venous thromboembolic disease present	Effect of Venous thromboembolic disease within the strata of Epilepsy
	OR [95% CI]	OR [95% CI]	OR [95% CI]
Epilepsy absent	1 [Reference]	1.11 [1.04, 1.18]	1.11 [1.04, 1.18]
Epilepsy present	1.36 [1.19, 1.54]	2.67 [1.74, 4.09]	1.97 [1.26, 3.06]
Effect of Epilepsy within the strata of Venous thromboembolic disease	1.36 [1.19, 1.54]	2.4 [1.56, 3.69]	
Age	1.05 [1.05, 1.06]		
Multiplicative scale	1.77 [1.13, 2.77]		
RERI	**1.2 [0.26, 2.63]**		

7k: Osteoarthritis and Stroke in men, model adjusted for age

*	Stroke absent	Stroke present	Effect of Stroke within the strata of Osteoarthritis
	OR [95% CI]	OR [95% CI]	OR [95% CI]
Osteoarthritis absent	1 [Reference]	1.52 [1.4, 1.65]	1.52 [1.4, 1.65]
Osteoarthritis present	1.25 [1.18, 1.32]	2.84 [2.31, 3.5]	2.27 [1.84, 2.81]
Effect of Osteoarthritis within the strata of Stroke	1.25 [1.18, 1.32]	1.87 [1.5, 2.33]	
Age	1.04 [1.04, 1.04]		
Multiplicative scale	1.49 [1.19, 1.87]		
RERI	**1.07 [0.52, 1.73]**		

7l: Connective tissue disease and Diabetes in men, model adjusted for age

*	Diabetes absent	Diabetes present	Effect of Diabetes within the strata of Connective tissue disease
	OR [95% CI]	OR [95% CI]	OR [95% CI]
Connective tissue disease absent	1 [Reference]	1.61 [1.53, 1.69]	1.61 [1.53, 1.69]
Connective tissue disease present	2.74 [2.47, 3.04]	3.99 [3.11, 5.12]	1.46 [1.11, 1.9]
Effect of Connective tissue disease within the strata of Diabetes	2.74 [2.47, 3.04]	2.48 [1.93, 3.19]	
Age	1.04 [1.04, 1.04]		
Multiplicative scale	0.9 [0.69, 1.19]		
RERI	0.64 [-0.29, 1.8]		

7m: Diabetes and Stroke in men, model adjusted for age

*	Stroke absent	Stroke present	Effect of Stroke within the strata of Diabetes
	OR [95% CI]	OR [95% CI]	OR [95% CI]
Diabetes absent	1 [Reference]	1.52 [1.39, 1.66]	1.52 [1.39, 1.66]
Diabetes present	1.55 [1.48, 1.63]	2.87 [2.46, 3.35]	1.85 [1.58, 2.16]
Effect of Diabetes within the strata of Stroke	1.55 [1.48, 1.63]	1.89 [1.59, 2.25]	
Age	1.04 [1.04, 1.04]		
Multiplicative scale	1.22 [1.02, 1.46]		
RERI	**0.8 [0.37, 1.29]**		

7n: Diabetes and Uncorrectable vision problems in men, model adjusted for age

*	Uncorrectable vision problems absent	Uncorrectable vision problems present	Effect of Uncorrectable vision problems within the strata of Diabetes
	OR [95% CI]	OR [95% CI]	OR [95% CI]
Diabetes absent	1 [Reference]	1.03 [0.93, 1.14]	1.03 [0.93, 1.14]
Diabetes present	2.6 [2.22, 3.05]	1.53 [1.37, 1.7]	0.59 [0.52, 0.67]
Effect of Diabetes within the strata of Uncorrectable vision problems	2.6 [2.22, 3.05]	1.49 [1.42, 1.56]	
Age	1.04 [1.04, 1.04]		
Multiplicative scale	0.57 [0.48, 0.68]		
RERI	-1.1 [-1.53, -0.75]		

7o: Drug and/or alcohol misuse and Osteoarthritis in men, model adjusted for age

*	Osteoarthritis absent	Osteoarthritis present	Effect of Osteoarthritis within the strata of Drug and/or alcohol misuse
	OR [95% CI]	OR [95% CI]	OR [95% CI]
Drug and/or alcohol misuse absent	1 [Reference]	1.24 [1.18, 1.31]	1.24 [1.18, 1.31]
Drug and/or alcohol misuse present	1.34 [1.06, 1.7]	5.49 [3.32, 9.09]	4.1 [2.35, 7.14]
Effect of Drug and/or alcohol misuse within the strata of Osteoarthritis	1.34 [1.06, 1.7]	4.42 [2.67, 7.32]	
Age	1.04 [1.04, 1.04]		
Multiplicative scale	3.3 [1.89, 5.76]		
RERI	**3.91 [1.71, 7.51]**		

7p: Epilepsy and Stroke in men, model adjusted for age

*	Stroke absent	Stroke present	Effect of Stroke within the strata of Epilepsy
	OR [95% CI]	OR [95% CI]	OR [95% CI]
Epilepsy absent	1 [Reference]	1.53 [1.41, 1.66]	1.53 [1.41, 1.66]
Epilepsy present	1.51 [1.31, 1.74]	2.81 [2.07, 3.8]	1.86 [1.33, 2.6]
Effect of Epilepsy within the strata of Stroke	1.51 [1.31, 1.74]	1.83 [1.34, 2.51]	
Age	1.04 [1.04, 1.04]		
Multiplicative scale	1.22 [0.86, 1.71]		
RERI	0.77 [-0.01, 1.79]		

7q: Connective tissue disease and Osteoporosis in men, model adjusted for age

*	Osteoporosis absent	Osteoporosis present	Effect of Osteoporosis within the strata of Connective tissue disease
	OR [95% CI]	OR [95% CI]	OR [95% CI]
Connective tissue disease absent	1 [Reference]	1.86 [1.57, 2.21]	1.86 [1.57, 2.21]
Connective tissue disease present	2.51 [2.28, 2.77]	5.64 [3.43, 9.27]	2.24 [1.35, 3.72]
Effect of Connective tissue disease within the strata of Osteoporosis	2.51 [2.28, 2.77]	3.03 [1.8, 5.12]	
Age	1.04 [1.04, 1.04]		
Multiplicative scale	1.21 [0.71, 2.05]		
RERI	**2.27 [0.02, 5.91]**		

7r: Coronary artery disease and Stroke in men, model adjusted for age

*	Stroke absent	Stroke present	Effect of Stroke within the strata of Coronary artery disease
	OR [95% CI]	OR [95% CI]	OR [95% CI]
Coronary artery disease absent	1 [Reference]	1.45 [1.32, 1.59]	1.45 [1.32, 1.59]
Coronary artery disease present	1.18 [1.12, 1.24]	2.2 [1.92, 2.53]	1.87 [1.62, 2.15]
Effect of Coronary artery disease within the strata of Stroke	1.18 [1.12, 1.24]	1.52 [1.29, 1.79]	
Age	1.04 [1.04, 1.04]		
Multiplicative scale	1.29 [1.09, 1.53]		
RERI	**0.58 [0.26, 0.92]**		

7s: Diabetes and Osteoarthritis in men, model adjusted for age

*	Osteoarthritis absent	Osteoarthritis present	Effect of Osteoarthritis within the strata of Diabetes
	OR [95% CI]	OR [95% CI]	OR [95% CI]
Diabetes absent	1 [Reference]	1.3 [1.23, 1.38]	1.3 [1.23, 1.38]
Diabetes present	1.59 [1.51, 1.67]	2.2 [1.97, 2.47]	1.38 [1.23, 1.56]
Effect of Diabetes within the strata of Osteoarthritis	1.59 [1.51, 1.67]	1.69 [1.5, 1.91]	
Age	1.04 [1.04, 1.04]		
Multiplicative scale	1.06 [0.93, 1.21]		
RERI	**0.31 [0.06, 0.58]**		

OR: odds ratio. CI: confidence interval

RERI: Relative excess risk of interaction. The difference between the observed relative risk and the combined relative risk of the two conditions individually on the additive scale.

Supplementary table 8: Odds ratios of sarcopenia for those single and three condition combinations identified in decision tree analyses. Estimates shown compare those people with the condition to those people without (ref) in age adjusted models.

	**OR [95% CI]**
**Women**	
Connective tissue disease	2.91 [2.74, 3.09]
Age	1.05 [1.05, 1.06]
Coronary artery disease	1.35 [1.26, 1.44]
Diabetes	1.29 [1.22, 1.37]
Uncorrectable vis	0.93 [0.84, 1.02]
Age	1.05 [1.05, 1.05]
Coronary artery disease and Diabetes	1.04 [0.88, 1.22]
Coronary artery disease and Uncorrectable vision problems	1.27 [0.88, 1.82]
Diabetes and Uncorrectable vision problems	1.45 [1.18, 1.78]
Coronary artery disease and Diabetes and Uncorrectable vision problems	1.43 [0.80, 2.60]
**Men**	
Connective tissue disease	2.56 [2.33, 2.82]
Age	1.04 [1.04, 1.04]
Paralysis	1.59 [1.37, 1.85]
Age	1.04 [1.04, 1.04]
Osteoporosis	2.00 [1.70, 2.35]
Age	1.04 [1.04, 1.04]

OR: odds ratio. CI: confidence interval
