# Supplementary material for: Identifying combinations of long-term conditions associated with sarcopenia: a cross-sectional decision tree analysis in the UK Biobank study
Source: BMJ Open. 2024 Sep 5;14(9):e085204. doi: 10.1136/bmjopen-2024-085204 (PMC11381693; doi:10.1136/bmjopen-2024-085204)
Supplement: Supplementary file 2 [file bmjopen-2024-085204.reviewer_comments.pdf]

PEER REVIEW HISTORY

BMJ Open publishes all reviews undertaken for accepted manuscripts. Reviewers are asked to complete a checklist review form and are provided with free text boxes to elaborate on their assessment. These free text comments are reproduced below.

ARTICLE DETAILS

**TITLE (PROVISIONAL)**	Identifying combinations of long-term conditions associated with sarcopenia: a cross-sectional decision tree analysis in the UK Biobank study
**AUTHORS**	Hillman, Susan; Dodds, Richard; Granic, Antoneta; Witham, Miles; Sayer, Avan; Cooper, Rachel

VERSION 1 - REVIEW

**REVIEWER NAME**	Hong, Namki
**REVIEWER AFFILIATION**	Yonsei University College of Medicine
**REVIEWER CONFLICT OF INTEREST**	I have no competing interests regarding this study.
**DATE REVIEW RETURNED**	06-Mar-2024

**GENERAL COMMENTS**	Title: Can machine learning identify combinations of long-term conditions associated with increased sarcopenia risk in people with multimorbidity? Findings from UK Biobank<br><br>Narrative review: Authors analyzed the association between long-term conditions and presence of sarcopenia using a subset of UK Biobank dataset. By applying decision tree analysis, several conditions were found to be associated with presence of sarcopenia (at risk of sarcopenia). Although this study is clinically relevant, I have some comments that may help to improve the manuscript.<br><br>Major comments:<br>1) Although authors emphasized the use of machine learning in the title, it is hard to find how the decision tree models were built and fine-tuned. Hyperparameters for the models are also missing. Rationals for choosing decision tree, among several ML algorithms that may fit the purpose of the study, remains unclear.<br>2) Contribution of the features (or importance of features) can vary sensitively by the details of the ML models. Therefore, outcome from a single ML model needs to be further validated and replicated by other ML models when one wanted to see the relative contribution of features (long-term conditions) to the prediction (presence of sarcopenia in this case). Perhaps long-term conditions that are commonly supported by various ML algorithms as major contributing features repeatedly would be more reliable for the study purpose. Relative quantification of feaSHapley Additive exPlanations (SHAP)<br>3) One of the main outcome is relative excess risk of interaction of various diseases in association with sarcopenia. However, these results were presented in the discussion part and supplementary material but not in the main result. I would suggest authors to reorganize main and supplemental tables (and reduce some of supplemental tables that are not essential to the study) so that readers can focus on main findings of the study. It is hard to find the novelty and strength of findings in current form.

	Minor comments:<br>1) This study is cross-sectional study that analyzed the association between long-term conditions and presence of sarcopenia (possible sarcopenia). Please consider revising the term ‘risk [indicating new-onset disease during follow-up]’ to ‘odds’ or ‘presence of sarcopenia’.

**REVIEWER NAME**	Yuan, Huaihong
**REVIEWER AFFILIATION**	Sichuan University West China Hospital
**REVIEWER CONFLICT OF INTEREST**	None.
**DATE REVIEW RETURNED**	07-Mar-2024

**GENERAL COMMENTS**	The writing of this study is quite comprehensive and meticulous. The authors have presented an excellent discussion of the research methods, findings, limitations, and significance. Overall, through its innovative approach, valuable discoveries, and insights for future research directions, this article has made an important contribution to the fields of epidemiology and geriatric medicine.

**REVIEWER NAME**	Özgür, Su
**REVIEWER AFFILIATION**	Ege Universitesi
**REVIEWER CONFLICT OF INTEREST**	None.
**DATE REVIEW RETURNED**	11-Mar-2024

**GENERAL COMMENTS**	First and foremost, I would like to extend my congratulations to the team that accessed and evaluated such a vast database. The dataset boasts a notably large sample size, making it crucial to acknowledge and emphasize the significance of studies conducted with existing data. Therefore, I extend my gratitude to you.<br><br>Sarcopenia is a disease of significant importance, requiring clarification of various details in clinical settings; hence, early diagnosis holds paramount importance. The decision tree method proves suitable for analyzing such extensive datasets, with adjustments made in the models to account for confounding factors. Sensitivity analysis results have been presented, with separate models studied for men and women, thereby ensuring a well-structured study design. Some minor corrections are necessary for the work.<br><br>Please revise page 9, line 34, where the Wilcoxon rank test is mentioned. Instead, the Mann-Whitney U test should be employed for comparing repeated nonparametric continuous variables (before and after). This test is suitable for two independent continuous variables that are not normally distributed.<br>Additionally, on page 12, line 58, for Tuberculosis, please present the p-value as 0.999 instead of 1.000.<br>It would be beneficial to include the results of Sensitivity and Accuracy values in table 3.<br>Lastly, ensure the full version of abbreviations such as OR (Odds Ratio) and CI (Confidence Interval) are provided in the tables below the respective tables."

VERSION 1 – AUTHOR RESPONSE

Reviewer 1

Narrative review: Authors analyzed the association between long-term conditions and presence of sarcopenia using a subset of UK Biobank dataset. By applying decision tree analysis, several conditions were found to be associated with presence of sarcopenia (at risk of sarcopenia). Although this study is clinically relevant, I have some comments that may help to improve the manuscript.

Response: We thank the reviewer for their positive assessment of our study and their constructive comments. In addressing these comments we believe that our manuscript has been strengthened.

Major comments:

1) Although authors emphasized the use of machine learning in the title, it is hard to find how the decision tree models were built and fine-tuned. Hyperparameters for the models are also missing. Rationals for choosing decision tree, among several ML algorithms that may fit the purpose of the study, remains unclear.

Response: We thank the reviewer for highlighting this and have amended our title in light of this comment, and a comment from the editor, to ensure it does not place undue emphasis on machine learning.

At the reviewer's request we have also ensured that our model hyperparameters are explicitly stated in the Methods section (page 8).

To clarify our rationale for using decision trees we have revised the relevant section of the introduction (page 4).

2) Contribution of the features (or importance of features) can vary sensitively by the details of the ML models. Therefore, outcome from a single ML model needs to be further validated and replicated by other ML models when one wanted to see the relative contribution of features (long-term conditions) to the prediction (presence of sarcopenia in this case). Perhaps long-term conditions that are commonly supported by various ML algorithms as major contributing features repeatedly would be more reliable for the study purpose. Relative quantification of feaSHapley Additive exPlanations (SHAP)

Response: We agree with the reviewer that the models presented must be validated and replicated. It was with this in mind that we aligned our results from decision tree analyses within an epidemiological framework by using the output of our decision tree analysis to undertake subsequent analyses using logistic regression models. This improves confidence in our findings and acts as a form of validation. It has the added benefit of providing results including odds ratios and information on interactions that are more widely understood by the intended readership.

We have also conducted sensitivity analyses which further enhance confidence in the robustness of our findings.

3) One of the main outcome is relative excess risk of interaction of various diseases in association with sarcopenia. However, these results were presented in the discussion part and supplementary material but not in the main result. I would suggest authors to reorganize main and supplemental tables (and reduce some of supplemental tables that are not essential to the study) so that readers can focus on main findings of the study. It is hard to find the novelty and strength of findings in current form.

Response: Thank you for this comment. Although we believe our findings in relation to interactions are of interest, the major emphasis of our study was the identification of combinations of LTC most strongly associated with an increased probability of developing sarcopenia using decision tree analysis; with a subsequent examination using logistic regression to support our findings through triangulation. Outcomes relating to interactions are thus not the primary focus of this paper. Nonetheless we agree with the reviewer that more detail about this should be included in the results and so have now moved more detail to the results section (page 15). All the relevant outputs from these models, which it is advised are presented to allow the reader to fully interpret them (see Knol et al, International Journal of Epidemiology, 2012), are too extensive to be permitted in the main manuscript. Therefore, after careful consideration we have decided that retention of the supplementary material is justified for completeness and transparency of reporting.

Minor comments:

1) This study is cross-sectional study that analyzed the association between long-term conditions and presence of sarcopenia (possible sarcopenia). Please consider revising the term 'risk [indicating new-onset disease during follow-up]' to 'odds' or 'presence of sarcopenia'.

Response: We thank the reviewer for this comment which highlights the important need to provide clarification. We have used the term 'at risk of sarcopenia' to describe those people whose grip strength is less than two standard deviations below the mean for young adults. This therefore includes not only people with sarcopenia, but also those considered to have a greater probability of developing sarcopenia at a later time. This measure does not define sarcopenia prevalence at baseline however but represents a broader classification. We have clarified our use of this term in the Methods/Assessment of grip strength section (page 6).

Reviewer 2

The writing of this study is quite comprehensive and meticulous. The authors have presented an excellent discussion of the research methods, findings, limitations, and significance. Overall, through its innovative approach, valuable discoveries, and insights for future research directions, this article has made an important contribution to the fields of epidemiology and geriatric medicine.

Response: We thank the reviewer for their positive assessment of our study.

Reviewer 3

First and foremost, I would like to extend my congratulations to the team that accessed and evaluated such a vast database. The dataset boasts a notably large sample size, making it crucial to acknowledge and emphasize the significance of studies conducted with existing data. Therefore, I extend my gratitude to you.

Sarcopenia is a disease of significant importance, requiring clarification of various details in clinical settings; hence, early diagnosis holds paramount importance. The decision tree method proves suitable for analyzing such extensive datasets, with adjustments made in the models to account for confounding factors. Sensitivity analysis results have been presented, with separate models studied for men and women, thereby ensuring a well-structured study design. Some minor corrections are necessary for the work.

Response: We thank the reviewer for their positive assessment of our work and for their helpful suggestions.

Please revise page 9, line 34, where the Wilcoxon rank test is mentioned. Instead, the Mann-Whitney U test should be employed for comparing repeated nonparametric continuous variables (before and after). This test is suitable for two independent continuous variables that are not normally distributed.

Response: We apologise for the confusion caused with the naming of this statistical test. The test we used is equivalent to the Mann-Whitney U test, and so we have amended our manuscript so that it now refers to the Mann-Whitney U test (page 8 and table 1).

Additionally, on page 12, line 58, for Tuberculosis, please present the p-value as 0.999 instead of 1.000.

Response: Thank you for highlighting this, we have made this amendment.

It would be beneficial to include the results of Sensitivity and Accuracy values in table 3.

Response: We have added estimates of Sensitivity and Accuracy to table 3.

Lastly, ensure the full version of abbreviations such as OR (Odds Ratio) and CI (Confidence Interval) are provided in the tables below the respective tables.

Response: We have made these changes in all relevant tables.

VERSION 2 – REVIEW

**REVIEWER NAME**	Özgür, Su
**REVIEWER AFFILIATION**	Ege Universitesi
**REVIEWER CONFLICT OF INTEREST**	None.
**DATE REVIEW RETURNED**	12-May-2024

**GENERAL COMMENTS**	That's a really nice study. I congrats the team. Thanks for the revisions.<br>Best regards.
